# Hypertonicity-enforced BCL-2 addiction unleashes the cytotoxic potential of death receptors

**DOI:** 10.1038/s41388-018-0265-5

**Published:** 2018-04-30

**Authors:** Simon Sirtl, Gertrud Knoll, Dieu Thuy Trinh, Isabell Lang, Daniela Siegmund, Stefanie Gross, Beatrice Schuler-Thurner, Patrick Neubert, Jonathan Jantsch, Harald Wajant, Martin Ehrenschwender

**Affiliations:** 1 Institute of Clinical Microbiology and Hygiene, University Hospital Regensburg, Franz-Josef-Strauss-Allee 11, Regensburg, 93053 Germany; 2 Division of Molecular Internal Medicine, Medical Clinic and Polyclinic II, University Hospital Würzburg, Röntgenring 11, Würzburg, 97070 Germany; 3 Department of Dermatology, Friedrich-Alexander-University Erlangen-Nürnberg (FAU), Ulmenweg 18, Erlangen, 91054 Germany

## Abstract

Attempts to exploit the cytotoxic activity of death receptors (DR) for treating cancer have thus far been disappointing. DR activation in most malignant cells fails to trigger cell death and may even promote tumor growth by activating cell death-independent DR-associated signaling pathways. Overcoming apoptosis resistance is consequently a prerequisite for successful clinical exploitation of DR stimulation. Here we show that hyperosmotic stress in the tumor microenvironment unleashes the deadly potential of DRs by enforcing BCL-2 addiction of cancer cells. Hypertonicity robustly enhanced cytotoxicity of tumor necrosis factor (TNF)-related apoptosis-inducing ligand (TRAIL) and other DR ligands in various cancer entities. Initial events in TRAIL DR signaling remained unaffected, but hypertonic conditions unlocked activation of the mitochondrial death pathway and thus amplified the apoptotic signal. Mechanistically, we demonstrate that hyperosmotic stress imposed a BCL-2-addiction on cancer cells to safeguard the integrity of the outer mitochondrial membrane (OMM), essentially exhausting the protective capacity of BCL-2-like pro-survival proteins. Deprivation of these mitochondrial safeguards licensed DR-generated truncated BH3-interacting domain death agonist (tBID) to activate BCL-2-associated X protein (BAX) and initiated mitochondrial outer membrane permeabilization (MOMP). Our work highlights that hyperosmotic stress in the tumor environment primes mitochondria for death and lowers the threshold for DR-induced apoptosis. Beyond TRAIL-based therapies, our findings could help to strengthen the efficacy of other apoptosis-inducing cancer treatment regimens.

## Introduction

Death receptors (DR) stand out of the other tumor necrosis factor (TNF)-receptor superfamily members due to their capability to induce regulated forms of cell death (apoptosis and/or necroptosis). The discovery that DRs such as CD95 and TNF-related apoptosis-inducing receptor 1 (TRAIL-R1) and TRAIL-R2 are expressed on malignant cells rendered DRs a potential target in cancer therapy and spurred in-depth investigations of DR signaling networks [1–4]. Upon activation, the DRs CD95, TRAIL-R1, and TRAIL-R2 assemble a death-inducing signaling complex (DISC) to promote caspase-8 activation, the starting point of the extrinsically triggered apoptotic cascade. Caspase-8 promotes apoptosis either in a straightforward manner through robust activation of the caspase-3 (type-I cells), directly heralding the execution phase of apoptosis. Alternatively, active caspase-8 cleaves the BH3-interacting domain death agonist (BID) to truncated BID (tBID), which in turn stimulates BCL-2-associated X protein (BAX) and BCL-2-antagonist/killer (BAK) activity [5, 6]. Subsequent mitochondrial outer membrane permeabilization (MOMP) releases cytochrome c and second mitochondria-derived activator of caspases (SMACs), triggering assembly of the caspase-9-activating apoptosome and antagonizing anti-apoptotic inhibitor of apoptosis (IAP) proteins, respectively. Both events cooperate in caspase-3 activation and thus propagate cell death in a type-II mode.

Translating early in vitro and in vivo findings into strategies for DR-directed cancer therapy faces major challenges. Fulminant liver toxicity of CD95 agonists precluded further clinical evaluation [7, 8]. TRAIL, the cognate ligand of TRAIL-R1 and –R2, potently killed cancer cells without lethal adverse effects [3, 4], but TRAIL-based therapies thus far failed in clinical trials [9]. The latter was (among others) attributed to insufficient potency of the drug candidates to activate TRAIL DRs and resistance of many primary tumors to TRAIL-induced apoptosis [10]. Several cell intrinsic factors contribute to apoptosis resistance, e.g., high levels of anti-apoptotic proteins. Notably, a pivotal role for the tumor microenvironment is also emerging [11]. We previously reported that the hypoxic tumor environment regulates TRAIL sensitivity in colorectal cancer cells through mitochondrial autophagy [12]. Here we show that hyperosmotic stress in the tumor environment robustly enhances cytotoxicity of TRAIL and other DR ligands in various cancer entities. Early events in TRAIL DR signaling remained unaffected, but hypertonic conditions amplified the DR-triggered apoptotic signal by unlocking tBID-mediated activation of the mitochondrial death pathway. Hyperosmotic stress imposed a BCL-2 addiction on cancer cells to safeguard the integrity of the outer mitochondrial membrane (OMM). This overburdened the remaining protective capacity of BCL-2-like pro-survival proteins to neutralize DISC-generated tBID, which in turn activated BAX and initiated MOMP. Mechanistically, our work identifies the osmotic pressure in the tumor microenvironment as a biophysical factor that affects mitochondrial priming and thus modulates the threshold for DR-induced apoptosis. Beyond TRAIL-based therapies, our findings could help to strengthen the efficacy of other apoptosis-inducing cancer treatment regimens.

## Results

### Hypertonic conditions robustly enhance DR-induced apoptosis

**Fig. 1:**
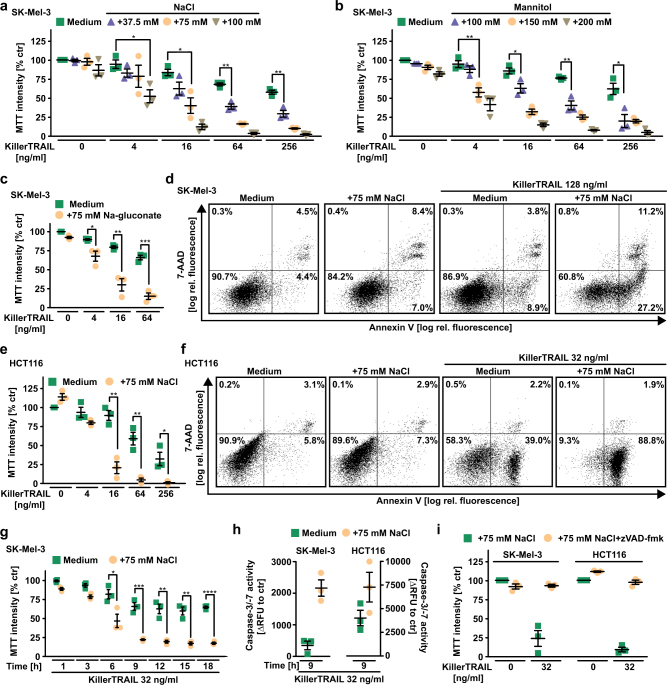
Hyperosmotic stress enhances TRAIL-induced apoptosis. **a**–**c** and **e** SK-Mel-3 and HCT116 cells were challenged with the indicated concentrations of KillerTRAIL in the presence and absence of the indicated concentrations of **a**, **e** NaCl, **b** mannitol, and **c** sodium gluconate. **d** and **f** SK-Mel-3 and HCT116 cells were challenged with the indicated concentrations of KillerTRAIL for 6 h in the presence and absence of NaCl (75 mM). Cells were subsequently analyzed by flow cytometry for 7-AAD- and annexin-V positivity. Data shown are representative of two experiments performed. **g** SK-Mel-3 cells were challenged with KillerTRAIL (32 ng/ml) for the indicated periods of time in the presence and absence of NaCl (75 mM). **h** SK-Mel-3 (left side) and HCT116 cells (right side) were treated with KillerTRAIL (32 ng/ml) for 9 h in the presence and absence of NaCl (75 mM). Caspase-3/-7 activity was assessed using the fluorogenic substrate (DEVD)_2_-R110. **i** SK-Mel-3 and HCT116 cells were challenged with KillerTRAIL (32 ng/ml) in the presence and absence of NaCl (75 mM) and zVAD-fmk (100 µM). With the exception of **d** and **f**, data points and mean ± SEM from three independent experiments are shown. *p ≤ 0.05, **p ≤ 0.01, ***p ≤ 0.001, *p < 0.0001, RFU, relative fluorescence units

**Fig. 2:**
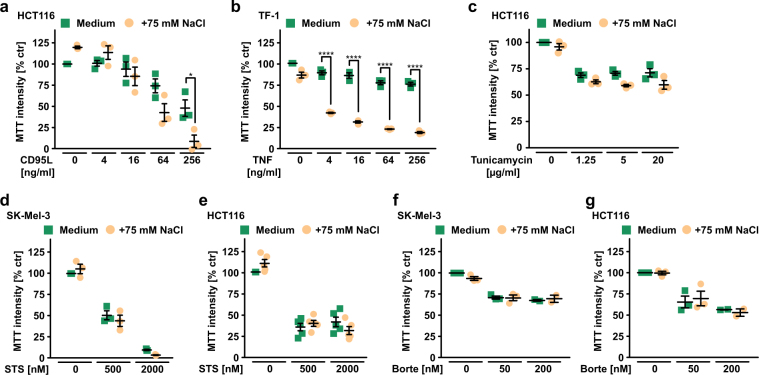
Hypertonicity specifically sensitizes to death receptor-mediated cytotoxicity. **a**–**g** HCT116, TF-1, and SK-Mel-3 cells were challenged with the indicated concentrations of **a** CD95L, **b** TNF, **c** tunicamycin, **d** and **e** staurosporine, and **f** and **g** bortezomib in the presence and absence of NaCl (75 mM). Shown are data points and mean ± SEM from three independent experiments. *p ≤ 0.05, **p ≤ 0.01, ***p ≤ 0.001, *p < 0.0001, Borte, bortezomib; STS, staurosporine

Exogenous addition or accumulation of osmotically active solutes that cannot passively diffuse across the plasma membrane (e.g., NaCl or mannitol) establishes an osmotic pressure gradient between the intra- and extracellular space (hyperosmotic stress or hypertonicity). Cellular adaption to hyperosmotic stress requires (among others) activation of nuclear factor of activated T-cells 5 (NFAT5), an essential transcription factor for upregulation of osmoprotective genes (Supplementary Figure S1a) [13]. Treatment of melanoma cells (SK-Mel-3) with an oligomerized and highly bioactive TRAIL variant (KillerTRAIL, hereafter referred to as TRAIL) displayed significantly enhanced killing when hypertonic conditions were generated by adding NaCl, mannitol, or sodium gluconate (Fig. 1a–c). All osmolytes exerted no significant cytotoxic effect per se, but drastically increased the percentage of 7-aminoactinomycin D- and/or annexin-V-positive cells upon TRAIL treatment compared with isotonic conditions (Fig. 1d). We confirmed the hypertonicity-granted boost in TRAIL cytotoxicity in other cell lines established from malignant melanoma (IGR-1 and A2058, Supplementary Figure S1b and c), colorectal cancer (HCT116, Fig. 1e,f and HT-29, Supplementary Figure S1d), acute lymphoblastic leukemia (REH), oral squamous cell carcinoma (PCI-68, Supplementary Figure S1e and f), and spheroid cultures (HCT116 spheroids, Supplementary Figure S1g) cells to exclude cell line-, tumor entity- or cell culture technique-specific phenomena. Robust cell death occurred ~ 6–9 h following TRAIL stimulation under hypertonic conditions (Fig. 1g) and (compared with isotonic controls) was accompanied by strikingly higher activation of the effector caspases 3 and 7 (Fig. 1h). Blocking caspase activation using the pan-caspase inhibitor zVAD-fmk (carbobenzoxy-valyl-alanyl-aspartyl-(Omethyl)-fluoromethylketone) completely rescued SK-Mel-3 and HCT116 from TRAIL-induced cytotoxicity cells in the presence of NaCl (Fig. 1i). Notably, we also observed enhanced cytotoxicity of CD95L and TNF (Fig. 2a,b) under hypertonic conditions, whereas toxicity of pharmacological cell death inducers such as tunicamycin, staurosporine, and bortezomib remained unchanged (Fig. 2c–g). Together, our data indicated that hyperosmotic stress enhanced DR-mediated apoptosis.

### Hypertonicity acts downstream of DISC formation to enhance TRAIL-induced apoptosis

**Fig. 3:**
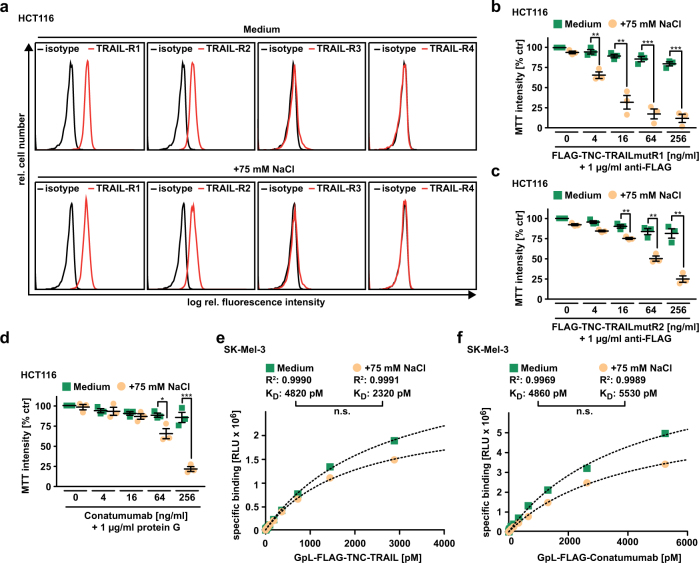
Changes in tonicity do not affect TRAIL-receptor expression and receptor-ligand interaction. **a** Cell surface expression of TRAIL-R1, TRAIL-R2, TRAIL-R3, and TRAIL-R4 was analyzed in HCT116 cells using flow cytometry. Data shown are representative of two experiments performed. **b** and **c** HCT116 cells were challenged with the indicated concentrations of **b** FLAG-TNC-TRAILmutR1 and **c** FLAG-TNC-TRAILmutR2 (both artificially cross-linked by adding anti-FLAG antibody) in the presence and absence of NaCl (75 mM). **d** HCT116 cells were treated with the indicated concentrations of the TRAIL-R2 targeting, agonistic antibody conatumumab (artificially cross-linked by adding protein G) in the presence and absence of NaCl (75 mM). For **b**–**d**, data points and mean ± SEM from three independent experiments are shown. **e** and **f** Equilibrium binding studies were performed by incubating SK-Mel-3 cells with the indicated concentrations of **e** GpL-FLAG-TNC-TRAIL and **f** GpL-FLAG-conatumumab as detailed in “Materials and methods.” Binding curves and *R*^2^ values shown are representative of three experiments performed; *K*_D_ values shown were calculated from three independent experiments. *p ≤ 0.05, **p ≤ 0.01, ***p ≤ 0.001, *p < 0.0001; n.s., not statistically significant; RLU, relative light units

In previous work, we demonstrated that hyperosmotic stress enhances cytotoxicity of SMAC mimetics (SMs) by increasing auto-/paracrine TNF secretion [14]. Adding TNF did not aggravate the cytotoxicity of TRAIL, whereas NaCl did (Supplementary Figure 2a and b). This excluded a pivotal role of hypertonicity-induced TNF production in TRAIL/NaCl-induced apoptosis. We next investigated the underlying molecular mechanism by assessing hypertonicity-associated alterations in the DR signaling cascade at various steps. Cell surface expression levels of TRAIL-R1 and -R2, as well as the decoy receptors TRAIL-R3 and -R4 were comparable in the presence and absence of NaCl in HCT116 (Fig. 3a) and SK-Mel-3 cells (Supplementary Figure S2c). TRAIL-R1- and TRAIL-R2-specific TRAIL constructs (TRAILmutR1 and TRAILmutR2) revealed that both TRAIL DRs displayed enhanced cytotoxicity under hypertonic conditions (Fig. 3b,c). Similarly, TRAIL-R2 activation using the TRAIL-R2-specific agonistic antibody conatumumab in the presence of NaCl enhanced TRAIL-R2-induced cell death, suggesting that the mode of TRAIL DR activation (ligand-mediated vs. agonistic antibody-mediated) was unimportant (Fig. 3d). In addition, equilibrium binding studies with *Gaussia princeps* luciferase (GpL)-tagged variants of TRAIL and conatumumab revealed no gross hypertonicity-induced changes in the equilibrium dissociation constant *K*_D_, a characteristic for receptor–ligand and receptor–agonist affinity (Fig. 3e,f). In the light of these findings, changes in TRAIL DR expression and/or ligand/agonist-binding affinity seemed unlikely to account for the hypertonicity-granted boost in TRAIL-induced apoptosis.

**Fig. 4:**
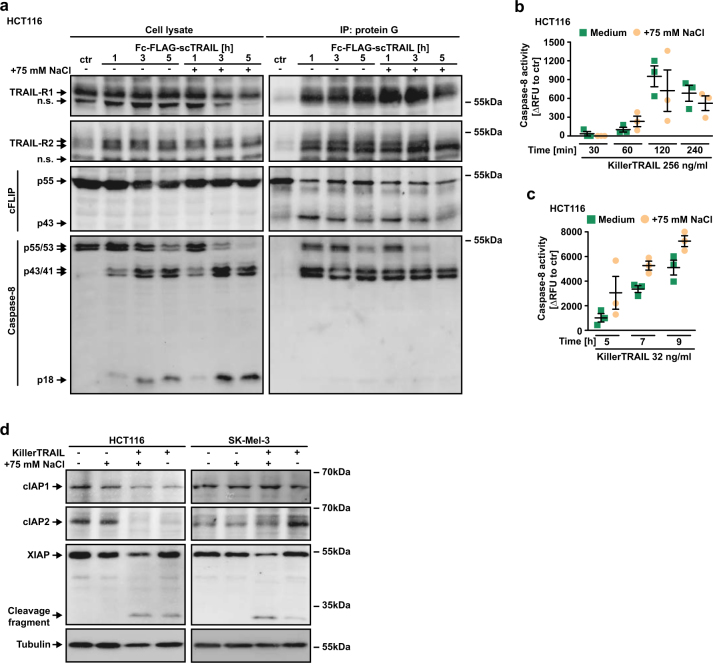
TRAIL-induced receptor signaling complex formation and caspase-8 activation is equally effective under iso- and hypertonic conditions. **a** TRAIL-R1/-R2 signaling complexes were induced in HCT116 cells by stimulation with Fc-FLAG-scTRAIL (1 μg/ml) for the indicated periods of time in the presence and absence of NaCl (75 mM). Proteins associated with Fc-FLAG-scTRAIL were immunoprecipitated using protein G agarose and were analyzed together with the corresponding lysates by western blotting for the presence of the indicated proteins. Data shown are representative of two experiments performed. **b** and **c** HCT116 cells were treated with KillerTRAIL (32 ng/ml) in the presence and absence of NaCl (75 mM) for the indicated periods of time. Caspase-8 activity was assessed using the fluorogenic substrate (IETD)_2_-R110. Please note that different concentrations of recombinant TRAIL required for caspase activity assays and co-immunoprecipitation experiments could affect the kinetics of caspase-8 activation. Shown are data points and mean ± SEM from three independent experiments. **d** HCT116 and SK-Mel-3 cells were challenged with KillerTRAIL (32 ng/ml) for 9 h in the presence and absence of NaCl (75 mM). After washing and lysis, western blot analyses were performed with antibodies specific for the indicated proteins. Detection of tubulin served as a loading control. RFU, relative fluorescence units

Immunoprecipitation experiments showed furthermore that TRAIL-induced recruitment of DISC components such as cFLIP and pro-caspase-8 occurred equally effective in isotonic and hypertonic conditions (Fig. 4a). DR-associated processing of caspase-8, indicative of DR-triggered caspase activation, was comparable in the absence and presence of NaCl (Fig. 4a, right panel) and correspondingly no hypertonicity-mediated enhancement in caspase-8 activity was observable at early time points after TRAIL challenge (Fig. 4b). Five hours post stimulation, an apparently higher amount of the active p18 fragment of caspase-8 was detectable in lysates of TRAIL/NaCl treated cells (Fig. 4a, left panel), concomitant with higher caspase-8 activity (Fig. 4c). This observation not necessarily reflects enhanced DR-mediated caspase-8 activation, but could also be attributable to increasing effector caspase-mediated caspase-8 activation in the course of already ongoing TRAIL/NaCl-induced apoptosis (Fig. 1g,h). Hypertonicity alone did not alter the cellular levels of the anti-apoptotic IAP family of proteins (Fig. 4d). In line with being caspase substrates [15, 16], cIAP1, cIAP2, and XIAP levels decreased proportionally to the strength (TRAIL vs. TRAIL/NaCl challenge) of the apoptotic stimulus (Fig. 4d). Thus far, our data suggested that TRAIL-induced signaling complex formation and initial caspase-8 activation was equally effective under iso- and hypertonic conditions.

### Hypertonicity depends on tBID-induced MOMP to enhance TRAIL-induced apoptosis

**Fig. 5:**
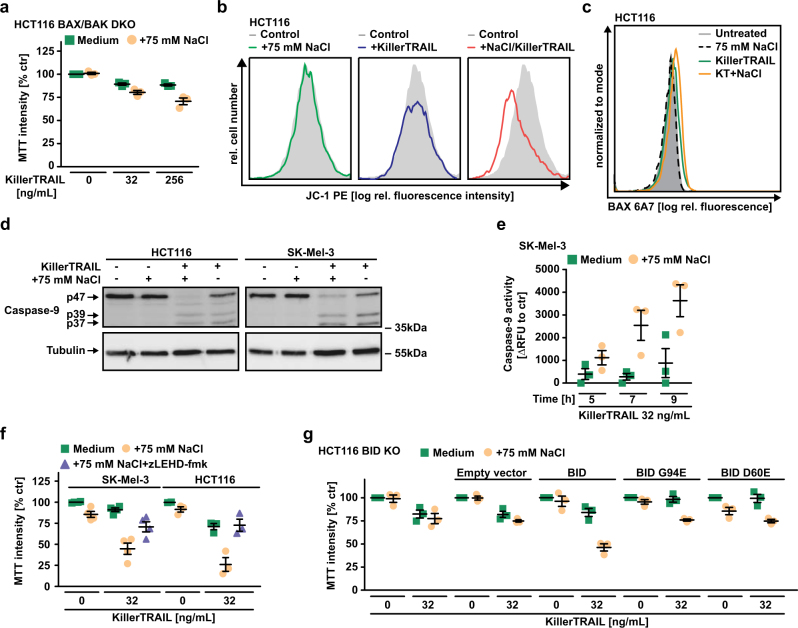
Hypertonicity licenses TRAIL to unlock MOMP. **a** HCT116 BAX/BAK double-knockouts were challenged with the indicated concentrations of KillerTRAIL in the presence and absence of NaCl (75 mM). **b** HCT116 cells were treated with NaCl (75 mM, first panel), KillerTRAIL (32 ng/ml, second panel), or a combination of both (third panel) for 3 h. Membrane potential of mitochondria was assessed by flow cytometry after staining with JC-1. **c** HCT116 cells were treated as in **b**. Conformational changes of BAX (indicative of ongoing activation) were measured using flow cytometry and the conformation-specific BAX antibody 6A7 [53]. Data shown for **b** and **c** are representative of three experiments performed. **d** HCT116 and SK-Mel-3 cells were challenged with KillerTRAIL (32 ng/ml) for 9 h in the presence and absence of NaCl (75 mM). After washing and lysis, western blot analyses were performed with antibodies specific for the indicated proteins. Detection of tubulin served as a loading control. Data shown are representative of two experiments performed. **e** SK-Mel-3 cells were treated with KillerTRAIL (32 ng/ml) in the presence and absence of NaCl (75 mM) for the indicated periods of time. Caspase-9 activity was assessed using the fluorogenic substrate (LEHD)_2_-R110. **f** SK-Mel-3 and HCT116 cells were challenged with the indicated concentrations of KillerTRAIL in the presence and absence of NaCl (75 mM) and the caspase-9 inhibitor zLEHD-fmk (50 µM). **g** HCT116 BID knockout cells or variants thereof complemented with empty vector, wild-type BID, BID G94E (mutated BH3 domain) or BID D60E (caspase-8 resistant) [50] were challenged with the indicated concentrations of KillerTRAIL in the presence and absence of NaCl (75 mM). For **a**, **e**–**g**, data points and mean ± SEM from three independent experiments are shown

Efficient DR-induced apoptosis in type-II cells (such as HCT116) requires BAX/BAK-mediated release of mitochondria-derived pro-apoptotic molecules. Deficiency in BAX/BAK or BAX alone attenuated or even abrogated TRAIL sensitivity under hypertonic conditions in HCT116 cells, whereas single loss of BAK was not protective (Fig. 5a and Supplementary Figure S3a-c). Apparently, MOMP remained a prerequisite for TRAIL DR-mediated apoptosis under hyperosmotic stress. In the presence of NaCl, TRAIL induced a rapid decrease in mitochondrial membrane potential (Fig. 5b) and activation-associated conformational changes in BAX (Fig. 5c and Supplementary Figure S3e), whereas TRAIL and NaCl alone had no effect. Concomitantly, NaCl increased TRAIL-induced caspase-9 processing and activity (Fig. 5d,e). Inhibiting caspase-9 using zLEDH-fmk completely rescued TRAIL/NaCl-challenged SK-Mel-3 and HCT116 cells (Fig. 5f). The extrinsic (DR-initiated) and intrinsic (mitochondria-mediated) apoptotic pathway are interlinked via the BH3-only protein BID. BID deficiency protected HCT116 cells from cell death upon TRAIL/NaCl challenge (Fig. 5g). Complementing HCT116 BID KO cells with wild-type BID but not with the caspase-8-resistant BID D60E or BH3 defective BID G94E mutant restored TRAIL-induced apoptosis under hypertonic conditions (Fig. 5g and Supplementary Figure S3d). In sum, hyperosmotic stress enhanced amplification of the TRAIL DR-derived death signal at the mitochondrial level via BID-induced, BAX-mediated MOMP with subsequent caspase-9 activation.

### Hyperosmotic stress enforces addiction to anti-apoptotic BCL-2 family proteins

**Fig. 6:**
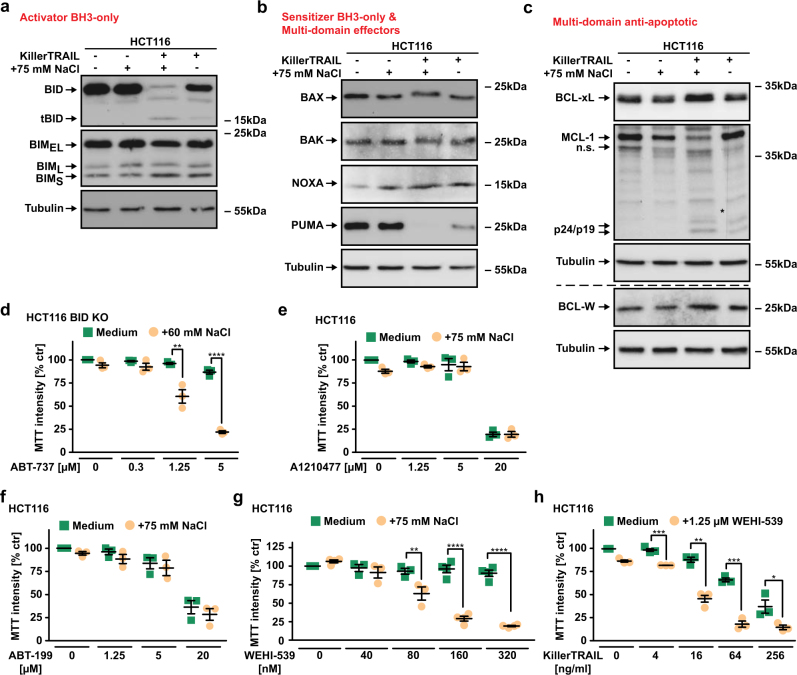
Hyperosmotic stress drives BCL-2 addiction of cancer cells. **a**–**c** HCT116 cells were challenged with KillerTRAIL (32 ng/ml) for 9 h in the presence and absence of NaCl (75 mM). After washing and lysis, western blot analyses were performed with antibodies specific for the indicated proteins. Detection of tubulin served as a loading control. The dashed line in **c** indicates that different cell lysates were used for BCL-W detection. The asterisk (*) in the MCL-1 blot indicates a defect in the CCD sensor of the western blot imaging system. All samples were run on the same gel, no gels were sliced. Data shown for **a**–**c** are representative of at least two experiments performed. **d**–**g** HCT116 and HCT116 BID knockout cells were challenged with the indicated concentrations of **d** ABT-737 (targeting BCL-2, BCL-xL, and BCL-W), **e** A1210477 (targeting MCL-1), **f** ABT-199 (targeting BCL-2), and **g** WEHI-539 (targeting BCL-xL) in the presence and absence of the indicated concentrations of NaCl. **h** HCT116 cells were challenged with the indicated concentrations of KillerTRAIL in the presence and absence of the BCL-xL inhibitor WEHI-539. For **d**–**h**, data points and mean ± SEM from three independent experiments are shown. *p ≤ 0.05, **p ≤ 0.01, ***p ≤ 0.001, *p < 0.0001

Caspase-mediated cleavage of BID and the integrity of its BH3 domain were indispensable for TRAIL/NaCl-induced killing of HCT116 cells (Fig. 5g). Hypertonicity could therefore enhance caspase-dependent tBID generation, a potent activator of BAX. Indeed, hyperosmotic stress enhanced loss of full-length BID upon TRAIL challenge (Fig. 6a), which is suggestive of caspase-8-mediated BID cleavage [17]. In our experimental setup, however, TRAIL-induced caspase-8 activation at early time points was comparable under iso- and hypertonic conditions (Fig. 4b), thus questioning whether the increase in BID cleavage is truly related to early TRAIL DR-associated events. Notably, caspase-3 is also capable to cleave BID [18] and is activated upon TRAIL/NaCl challenge (Fig. 1h). Enhanced TRAIL-induced BID cleavage under hypertonic conditions could therefore reflect already established MOMP and full-blown effector caspase activation rather than a hypertonicity-related boost in DISC-mediated tBID generation. Functionally, this raised the question at what level hypertonic conditions act to enforce the TRAIL DR-derived apoptotic signals in a mitochondria-dependent manner: (a) by enhancing DISC-mediated tBID generation or (b) by priming mitochondria for death downstream of tBID.

MOMP is governed by BCL-2 family interactions and primed mitochondria are dependent on anti-apoptotic members of the BCL-2 protein family to prevent loss of the mitochondrial membrane potential (BCL-2 addiction) [19]. Notably, total cellular levels of the different BCL-2 family subgroups (activator and sensitizer BH3-only, multi-domain anti-apoptotic, and multi-domain effectors) did not grossly change under hypertonic conditions (Fig. 6a–c and Supplementary Figure S4a and b), although ongoing apoptosis following TRAIL/NaCl challenge expectedly revealed caspase-dependent cleavage/degradation of BID, MCL-1 and PUMA [17, 20, 21]. In BID-deficient HCT116 cells, the BCL-2-, BCL-xL-, and BCL-W-antagonizing BH3-mimetic ABT-737 was only marginally toxic under isotonic conditions, but robustly triggered cell death upon the addition of NaCl (Fig. 6d). Selective targeting of MCL-1 (A1210477), BCL-2 (ABT-199), and BCL-xL (WEHI-539) revealed that hyperosmotic stress apparently enforced BCL-xL addiction of cancer cells to survive (Fig. 6e–g). Conversely, combinatorial treatment with TRAIL and the BCL-xL inhibitor WEHI-539 under normotonic conditions resulted in robust cell death (Fig. 6h). Collectively, our data suggested that hypertonicity-enforced addiction to the anti-apoptotic functions of BCL-2 family members primes cancer cells for TRAIL DR-mediated apoptosis.

### Hypertonicity enhances TRAIL responsiveness of patient-derived melanoma cells

**Fig. 7:**
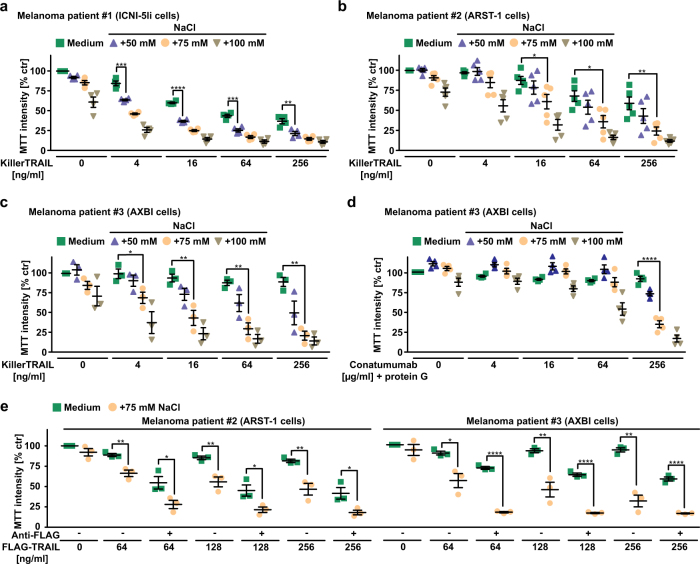
Hypertonicity enhances TRAIL responsiveness of patient-derived melanoma cells. **a**–**c** ICNI-5li (derived from patient 1), ARST-1 (derived from patient 2), and AXBI (derived from patient 3) cells were treated with the indicated concentrations of KillerTRAIL in the presence and absence of NaCl (50, 75, and 100 mM, respectively). **d** AXBI cells (derived from patient 3) were treated with the indicated concentrations of conatumumab (cross-linked with 1 µg/ml protein G). **e** ARST-1 and AXBI cells (derived from patients 2 and 3) cells were challenged with either soluble trimeric FLAG-TRAIL or oligomerized FLAG-TRAIL (through incubation with 1 µg/ml anti-FLAG antibody) in the presence and absence of the indicated concentrations of NaCl. Shown are data points and mean ± SEM from three independent experiments

Clinically, TRAIL-based therapies demonstrated a convincing safety profile but thus far displayed little success, which has (among others) been attributed to resistance of tumors to TRAIL-induced apoptosis and insufficient agonistic activity of currently available drug candidates in the clinic. We therefore next validated our findings in melanoma cells derived from three patients with metastatic disease. Hyperosmotic stress unequivocally enhanced cell death induced by TRAIL (Fig. 7a–c). Hypertonicity also boosted cytotoxicity of the TRAIL-R2 agonist conatumumab (Fig. 7d) and soluble (trimeric) FLAG-TRAIL (Fig. 7e). Not surprisingly, antibody-mediated cross-linking of the trimeric ligand enhanced bioactivity under iso- and hypertonic conditions.

**Fig. 8:**
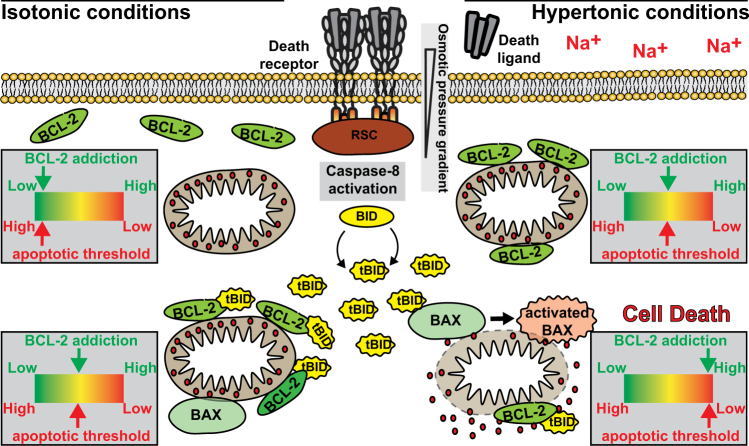
Hypertonicity-enforced Bcl-2 addiction unleashes the cytotoxic potential of death receptors in cancer cells. Left side: under isotonic conditions, BCL-2 addiction of cancer cells is low, yielding a high apoptotic threshold. Upon TRAIL DR activation, caspase-8 converts of BID into tBID. The potency of tBID to activate the pro-apoptotic effector BCL-2 protein family members BAX and BAK has to be counteracted by anti-apoptotic BCL-2-like proteins (such as BCL-2 itself or BCL-xL) to prevent MOMP. This augments BCL-2 addiction and lowers the apoptotic threshold, essentially priming mitochondria for death. However, the remaining capacity of anti-apoptotic BCL-2-family proteins is still sufficient to cope with TRAIL DR-triggered death signals, which in type-II cells effectively abrogates TRAIL DR-induced cell death. Right side: hypertonic conditions impose a higher degree of BCL-2 addiction on cancer cells, which lowers the apoptotic threshold even under “steady-state” conditions. The capacity of anti-apoptotic BCL-2-family proteins is sufficient to prevent hypertonicity-induced MOMP, but additional mitochondria-directed apoptotic stimuli (such as DISC-mediated tBID generation) rapidly exhaust the remaining ‘neutralization capacity’. This results in activation of BAX and MOMP, ultimately ending the cell’s life

In sum, we demonstrated that hyperosmotic stress enforced BCL-2 addiction of cancer cells and lowered the threshold for engagement of the mitochondrial cell death pathway. Death-primed mitochondria readily amplified TRAIL DR-derived apoptotic signals, which in type-II cells is a prerequisite for efficient apoptosis induction (summarized in Fig. 8).

## Discussion

TRAIL-based therapies are capable to specifically kill cancer cells without collateral damage in healthy tissue [3, 4]. Thus far, clinical trials largely failed as tumors evaded TRAIL-induced apoptosis and/or potency of TRAIL DR-activating molecules (soluble TRAIL, TRAIL-R1-, and TRAIL-R2-targeting agonistic antibodies) was modest [10]. Here we identify osmotic stress in the tumor microenvironment as a novel factor that aggravates DR-mediated apoptosis and boosts the deadly potential of “weak” TRAIL DR-targeting drug candidates. Mechanistically, hyperosmotic stress enforced BCL-2 addiction of cancer cells and thereby decreased the remaining capacity of mitochondria-safeguarding BCL-2-like proteins. Functionally, this lowered resistance of mitochondria to DR-generated OMM-targeting molecules such as tBID and thus unlocked amplification of the apoptotic signal. TRAIL DR-induced apoptosis under hypertonic conditions still followed a type-II mode (Fig. 5e–g), which contrasts other TRAIL DR-sensitizing strategies that enforced a switch to type-I mode of apoptosis [22, 23].

The modest potency of currently clinically used TRAIL-R1/-R2 agonists guarantees an excellent safety profile, but is obviously also a major cause for clinical failure. Substantial differences the cytotoxic activity of soluble trimeric TRAIL versus artificially oligomerized TRAIL variants and membrane TRAIL were early recognized [24–26]. Due to safety concerns, however, oligomerized forms of neither recombinant TRAIL nor TRAIL-R1/-R2-targeting agonists were moved forward to clinical development. A more potent “second-generation” of TRAIL and TRAIL-receptor agonists is under way [27], but will this be paid for with detrimental off-target effects? Notably, there are alternative strategies to boost the TRAIL DR-activating potential of available drug candidates. Soluble TRAIL and the TRAIL-R2 targeting antibody conatumumab synergistically induced apoptosis with good tolerability in vivo [28, 29]. This is most likely attributable to antibody-assisted secondary aggregation of initially formed trimeric receptor–ligand complexes to fully active receptor clusters, an essential step in TRAIL DR signaling [30]. We observed aggravated cytotoxicity of soluble trimeric TRAIL under hypertonic conditions in the absence of any cross-linking agent (Fig. 7e). Thus, hypertonicity enhanced TRAIL DR signaling either by facilitating spontaneous self-aggregation of trimeric TRAIL/TRAIL DR complexes into receptor clusters or lowered the threshold of cellular responses to weakly active trimeric receptor–ligand complexes. We provide evidence for the latter inasmuch as BCL-2 addiction under hypertonic conditions (Fig. 6d,g) lowers the threshold for tBID-mediated MOMP by overburdening the remaining BCL-2-like proteins (Fig. 6h). Substantial changes in the cellular levels of some BCL-2 family proteins were only detectable in the course on ongoing apoptosis, but not due to hyperosmotic stress alone (Fig. 6a–c and Supplementary Figure S4a and b). This was also observed in an earlier study that (somehow conflictingly) linked hypertonicity-induced TNF sensitization of HeLa cells to decreased BCL-2 protein levels in apoptotic cells [31].

A spatially restricted hypertonic tumor microenvironment could, on the one hand, complement existing strategies to enhance activity of TRAIL DR targeting agents and, on the other hand, limit efficient TRAIL DR activation to the tumor area. Acute onset of hypertonicity exceeding a certain level is sufficient to cause apoptotic cell death [32, 33]. It is therefore important to note that in our experimental setting hypertonicity alone had no or only modest toxic effects (Fig. 1a,e, Supplementary Figure S1, and Fig. 5b). Is it possible to establish an osmotic pressure gradient for the time needed to deliver/complete a treatment? For solid tumors, injection of non-diffusible osmolytes or their continuous release from implantable devices is technically feasible and from a therapeutic view may have implications beyond TRAIL-based cancer treatments. Many apoptosis inducing signals (including most chemotherapies) physically converge at the mitochondria, rendering the BCL-2-regulated (or intrinsic) apoptotic pathway associated with these organelles decisive whether to live or die [34]. This has stimulated development of BH3-mimetics (reviewed in Delbridge et al. [35]), a novel class of therapeutics that induces apoptosis or primes mitochondria for death by binding and inhibiting anti-apoptotic BCL-2 family proteins [19]. Mitochondrial priming is directly correlated with clinical response to cytotoxic chemotherapy [36]. Our finding that hypertonicity enforced BCL-2 addiction (and thus primed) cancer cells might therefore not only be of clinical relevance for TRAIL-based, but also other apoptosis-inducing cancer treatments.

Our experiments highlighted an essential role for BCL-xL (Fig. 6g), but not for BCL-2 and MCL-1 (Fig. 6e,f) to survive hypertonic conditions. BCL-xL retro-translocates BAX from the mitochondria into the cytosol and can sequester BAX-activating BH3-only proteins at subcellular membranes [37, 38]. BCL-xL also retro-translocates mitochondrial BAK (although significantly slower) [39], but HCT116 cells predominantly rely on BAX (and not the functionally redundant BAK) for apoptosis induction [40]. However, functions of anti-apoptotic BCL-2 family proteins can be at least partly redundant and dependency on BCL-xL or other safeguards might be cell-type specific. Nevertheless, this raises the question how hypertonicity enforces BCL-xL addiction in our experimental setup. Hyperosmotic stress enhanced activation of the effector protein BAX following TRAIL DR activation (Fig. 5c), but also upon ABT-737 treatment (data not shown). Perturbations in sensitizer and activator BH3-only proteins could be involved, although at least BID seems to be dispensable (Fig. 6d). In addition, MOMP in the absence of all BH3-only proteins has recently been demonstrated and in isolated mitochondria ionic strength ionic strength influences the dynamics of BAX channel formation [41, 42]. Hypertonicity-induced biophysical alterations in the cell could thus already be sufficient promote mitochondrial BAX translocation and subsequent activation, potentially facilitated by changing localization dynamics of BCL-xL to subcellular membranes [38]. Under otherwise steady-state conditions, BCL-xL still manages to safeguard the mitochondria, but is overburdened when challenged with BH3 mimetics or TRAIL DR-generated tBID (summarized in Fig. 8). Definitely, further work is required to solve these questions.

Beside cancer treatment, hypertonicity-induced mitochondrial priming might also affect immunomodulatory functions of TRAIL. TRAIL is expressed on various different immune cells such as monocytes, T cells, dendritic cells, and natural killer cells [43–46]. Recent studies elegantly demonstrated that hypertonic environments indeed occur in vivo (apart from the textbook example of the renal medulla). For example, bacterial skin infections in humans caused cutaneous Na^+^ accumulation (~ 40 mM Na^+^) and high dietary salt intake resulted in hyperosmolality in skin, muscle, and other tissues [47–49]. In is currently unclear whether “(patho-) physiologic hypertonicity” alters the response of TRAIL DR expressing non-malignant cells.

In sum, we provide evidence that tonicity of the cellular environment acts as a factor that co-determines the apoptotic threshold by modulating death priming of mitochondria. Our work adds another layer of complexity in the sophisticated web of TRAIL DR signaling.

## Material and methods

### Cell lines, antibodies, and chemicals

HCT116, HT-29, TF-1, and REH cells were obtained from the German Collection of Microorganisms and Cell Culture (Braunschweig, Germany). Sk-Mel-3 and IGR-1 cells were provided by Barbara Schmidt (University of Regensburg, Germany), A2058 cells by Jens Pietzsch (Helmholtz-Zentrum Dresden-Rossendorf, Germany), and authenticated using SNP-profiling (Multiplexion, Heidelberg, Germany). PCI-68 cells were a gift from Richard Bauer (University of Regensburg). BID-deficient HCT116 cells and the complemented variants thereof (HCT116 BID KO + empty vector, HCT116 BID KO + wt BID, HCT116 BID KO + BID D60E, HCT116 BID KO + BID G94E) were a kind gift from Xu Luo (University of Nebraska Medical Center, Nebraska, USA) [50]. HCT116 BAX/BAK DKO and HCT116 BAK KO cells were kindly provided by Richard Youle (National Institutes of Health, Bethesda, USA) [40]. HCT116 BAX KO cells were obtained from Bert Vogelstein (Johns Hopkins University, Baltimore, MA, USA) [51]. All cell lines were grown in RPMI 1640 medium (PAN Biotech, Aidenbach, Germany) supplemented with 10% (v/v) fetal calf serum (Sigma, Steinheim, Germany). AXBI, ARST-1, and ICNI-5li cell lines were (after informed consent) established from metastatic lesions of melanoma patients as described previously (approved by the ethics committee of the Medical Faculty, Friedrich-Alexander-Universität Erlangen-Nürnberg, EK 4602) [52]. These cell lines were grown in Dulbecco’s modified Eagle’s medium (Thermo Fisher, Waltham, MA, USA) supplemented with 10% (v/v) fetal calf serum (Sigma). HCT116 spheroids (3000 cells/spheroid) were grown in Nunclon Sphera 96-well plates (Thermo Fisher) for 72 h, according to manufacturer’s instructions, and subsequently challenged with KillerTRAIL for another 48 h. Viability of the spheroids was assessed using MTT (3-[4,5-dimethylthiazol-2-yl]-2,5-diphenyl tetrazolium bromide) staining. Antibodies used in the study were as follows: TRAIL-R1 (#854.853.020), TRAIL-R2 (#854.863.020) (Diaclone SAS, Besancon, France); cFLIP (#3210), caspase-8 (#9746), caspase-9 (#9502), cIAP1 (#7065), cIAP2 (#3130), XIAP (#2045), BID (#2002), BIM (#2933), BAX (#5023), BAK (#12105), PUMA (#12450), BCL-xL (#2764), MCL-1 (#5453), and BCL-W (#2724) (Cell Signaling, Beverly, MA, USA); tubulin (#MS-581) (Dunnlab, Asbach, Germany); conformation-specific [53] BAX (6A7, #sc-23959), NOXA (114C307, #sc-56169) (Santa Cruz, Santa Cruz, CA, USA); FLAG (M2, #F3165) (Sigma); NFAT5 (#PA1–023) (Thermo Fisher). Chemicals were as follows: MTT (Biomol, Hamburg, Germany); zVAD-fmk (Bachem, Bubendorf, Switzerland); zLEHD-fmk (BD Biosciences, Heidelberg, Germany); tunicamycin, ABT-737, ABT-199, A1210477, and WEHI-539 (Hycultec, Beutelsbach, Germany); staurosporine (Selleck Chemicals, Houston, TX, USA); and bortezomib (US Biological, Swampscott, MA, USA).

### Recombinant proteins

KillerTRAIL was purchased from Apronex (Jesenice u Prahy, Czech Republic), CD95L (ACRP30*headless*-CD95L, #AG-40B-0130-C010) and FLAG-TRAIL were purchased from Adipogen (Liestal, Switzerland). TNF was a kind gift from Daniela Männel (University of Regensburg). Production and purification of TRAIL variants harboring mutations conferring specificity for TRAIL-R1 (FLAG-TNC-TRAILmutR1, mutations G131R/R149I/S159R/N199R/K201H/S215D) [54] and TRAIL-R2 (FLAG-TNC-TRAILmutR2, mutations Y189Q/R191K/Q193R/H264R/I266L/D267Q) [55], and GpL-tagged variants thereof, was performed as described previously [56]. Expression plasmids encoding the heavy chain and light chain of conatumumab were derived from pCR3 (Invitrogen, Karlsruhe, Germany). The light-chain expression construct encodes an protein composed of the human IgG1 leader sequence followed by the amino acids QL, the FLAG epitope, the amino acids EL, the variable light chain sequence of conatumumab (KEGG database entry: D09329, light chain pos. 1–105; (http://www.genome.jp/kegg/)), the amino acid sequence GSEIKR and the amino acid sequence of the partial immunoglobulin κ-chain constant region (GenBank accession number: AAA58989.1). The heavy chain expression construct encodes an protein composed of the human IgG1 leader sequence followed by the amino acids QL, the FLAG epitope, the amino acids EL, the heavy chain sequence of conatumumab (KEGG database entry: D09329, heavy chain pos. 1–122), the amino acid sequence RSSS, and the amino acid sequence of the immunoglobulin heavy constant region γ1 (GenBank accession number P01857). Conatumumab was produced by co-transfection of light and heavy chain-encoding plasmids into HEK293 cells essentially as described elsewhere [57]. GpL-Conatumumab was obtained by co-transfection of the heavy chain-encoding plasmid and a variant of the light chain-encoding plasmid in which the amino acids LE and amino acids 18–185 of GpL (GenBank accession number GM037681) were inserted following the constant light chain part.

### MTT-based cell viability assay

Cells were seeded in 96-well plates (REH and TF-1 cells: 7 × 10^4^ cells/well; all other cell lines: 2 × 10^4^ cells/well) and challenged with the indicated concentrations of the indicated substances in duplicates (technical replicates). In case NaCl or other osmotically active solutes were used, these were simultaneously added. Unless indicated otherwise, cell viability was determined 18 h after stimulation using MTT staining (2 h at 37 °C). Staining intensity was measured at 595 nm and the mean was calculated from the technical replicates of each experiment. The mean value for untreated controls was set to 100%. For any other condition, the MTT staining intensity is given relative to the corresponding untreated group (% of control). Data points shown are mean values (calculated from two technical replicates) of independent experiments (*n* ≥ 3).

### Western blot analysis

Cells were collected, spun down, and were directly dissolved in 4 × Laemmli sample buffer (8% (w/v) SDS, 0.1 M dithiothreitol, 40% (v/v) glycerol, 0.2 M Tris, pH 8.0) supplemented with phosphatase inhibitor cocktails-I and -II (Sigma). Samples were sonicated and boiled for 5 min at 96 °C before proteins were separated by SDS-polyacrylamide gel electrophoresis and transferred to polyvinylidene difluoride membranes. To block nonspecific binding sites, membranes were incubated in Tris-buffered saline containing 0.1% (v/v) Tween 20 and 5% (w/v) dry milk before primary antibodies of the specificity of interest were added. Antigen-antibody complexes were visualized using horseradish peroxidase-conjugated secondary antibodies (Dako, Hamburg, Germany) and ECL technology (Pierce, Rockford, IL, USA).

### Caspase activity assays

Caspase activity was measured using the caspase-3/−7, caspase-9 and caspase-8 activity kit (AAT Bioquest, Sunnyvale, CA, USA) according to manufacturer’s instructions. Emitted fluorescence was quantified using a Victor3 Multilabel Reader (Perkin Elmer, Waltham, MA, USA).

### Flow cytometry

Cell death was assessed by annexin-V and 7-AAD staining. In brief, cells were challenged with the indicated concentrations of KillerTRAIL in the presence and absence of NaCl for 16 h. Afterwards, cells were stained with 7-AAD and annexin-V (4 °C for 15 min in the dark) following standard procedures [58], and analyzed immediately using a FACSCanto flow cytometer (BD Biosciences). For measuring cell surface expression of TRAIL-Receptors, cells were incubated for 30 min on ice with phycoerythrin (PE)-conjugated antibodies specific for the indicated TRAIL receptors (ProSci, Poway, CA, USA) or an appropriate isotype control (R&D Systems). Conformational changes in BAX indicating activation was measured as described previously [59]. Mitochondrial membrane potential was measured using the MitoScreen Kit (#551302, BD Biosciences) according to manufacturer’s instructions.

### Binding studies using GpL fusion proteins

Binding studies using the bioluminescent fusion proteins GpL–Flag–TNC–TRAIL and GpL-FLAG-conatumumab were essentially performed as described elsewhere [60]. Cells (typically 2 × 10^5^ per well in 24-well plates) were cultured overnight. For equilibrium binding experiments, cells were split in two groups to determine total and nonspecific binding. Cells of the “nonspecific binding” groups were pre-incubated with an excess of conventional TRAIL and conventional conatumumab (1 h at 37 °C), cells of the “total binding” groups remained untreated. Cells of both groups were subsequently pairwise incubated with increasing concentrations of GpL-Flag-TNC-TRAIL and GpL-FLAG-conatumumab for 1 h at 37 °C. Unbound ligand was removed by washing the plates 10 times for ~ 5 s in ice-cold phosphate-buffered saline. Next, cells were collected, resuspended in 50 µl RMPI 1640 medium (0.5% v/v fetal bovine serum), and transferred to black 96-well plates. GpL activity was quantified by measuring luminescence using the Gaussia luciferase assay kit (New England Biolabs, Frankfurt am Main, Germany) and a luminometer (AnthosLabtec Instruments, Krefeld, Germany). To obtain specific binding values, nonspecific binding values were subtracted from the corresponding total binding values. The maximum of specifically bound GpL activity (*B*_max_) and the dissociation constant (*K*_D_) were obtained by analysis of the specific binding values (non-linear regression analysis, one site specific binding model) using GraphPad Prism5 software (GraphPad Software, La Jolla, CA, USA).

### Co-immunoprecipitation

Immunoprecipitation was performed as described previously [12].

### Statistics

Unless otherwise specified, data are presented as individual data points from independent experiments and mean ± SEM. Comparisons were performed with a Student’s *t*-test whose values are denoted in the figures as *p ≤ 0.05, **p ≤ 0.01, ***p ≤ 0.001, and ****p < 0.0001.

## Electronic supplementary material

Supplementary Figures and Legends
