# Supplementary material for: Hypertonicity-enforced BCL-2 addiction unleashes the cytotoxic potential of death receptors
Source: Oncogene. 2018 Apr 30;37(30):4122–36. doi: 10.1038/s41388-018-0265-5 (PMC6062497; doi:10.1038/s41388-018-0265-5)
Supplement: Supplementary file 1 — Supplementary Figures and Legends [file 41388_2018_265_MOESM1_ESM.docx]

##

## Supplementary Figure S1: Hyperosmotic stress enhances TRAIL-induced apoptosis in various cancer cell lines.

(a) HCT116 and SK-Mel-3 cells were cultured in the presence and absence of NaCl (75 mM). After washing and lysis, Western blot analyses were performed with antibodies specific for the indicated proteins. Data shown are representative of 2 experiments performed. (b-f) IGR-1, A2058, HT-29, REH and PCI-68 cells were challenged with the indicated concentrations of KillerTRAIL in the presence and absence of the NaCl (75 mM). Shown are data points and mean±S.E.M. from three independent experiments. (g) HCT116 spheroids were challenged with KillerTRAIL (128 ng/ml) in the presence and absence of NaCl (75 mM) and subsequently stained with MTT (4 h). Data shown are representative of 3 experiments performed. *p ≤ 0.05, **p ≤ 0.01, ***p ≤ 0.001, *p < 0.0001.

##



## Supplementary Figure S2: Enhanced TRAIL cytotoxicity under hypertonic conditions is not attributable to TNF secretion or changes in TRAIL-receptor expression.

(a and b) HCT116 and SK-Mel-3 cells were challenged with the indicated concentrations of KillerTRAIL in the presence and absence of NaCl (75 mM) or TNF (200 ng/ml). Shown are data points and mean±S.E.M. from three independent experiments. (c) Cell surface expression of TRAIL-R1, TRAIL-R2, TRAIL-R3 and TRAIL-R4 was analyzed in HCT116 cells using flow cytometry. Data shown are representative of two experiments performed.

##



## Supplementary Figure S3: tBID-triggered MOMP is essential for hypertonicity-mediated enhancement of TRAIL-induced apoptosis.

(a) HCT116 BAX knockout cells and HCT116 BAK knockout cells were challenged with the indicated concentrations of KillerTRAIL in the presence and absence of NaCl (75 mM). Shown are data points and mean±S.E.M. from three independent experiments. (b) HCT116 BAX/BAK double-knockout cells were challenged with the indicated concentrations of KillerTRAIL for 6 h in the presence and absence of NaCl (75 mM). Cells were subsequently analyzed by flow cytometry for 7-AAD- and annexin-V-positivity. Data shown are representative of two experiments performed. (c) Lysates from HCT116 wild-type, BAX KO, BAK KO and BAX/BAK DKO were analyzed using Western blot with antibodies specific for the indicated proteins. (d) Lysates from HCT116 wild-type, BID KO and BID KO reconstituted with BID wt, BID G94E and BID D60E were analyzed using Western blot with antibodies specific for the indicated proteins. Detection of tubulin served as a loading control. Data shown are representative of two experiments performed.

##



## Supplementary Figure S4: Hyperosmotic stress does not change expression levels of BCL-2 family proteins.

(a and b) SK-Mel-3 cells were challenged with KillerTRAIL (32 ng/ml) for 9 h in the presence and absence of NaCl (75 mM). After washing and lysis, Western blot analyses were performed with antibodies specific for the indicated proteins. Detection of tubulin served as a loading control. The dashed line in (b) indicates that different cell lysates were used for BCL-2 detection. Data shown are representative of at least two experiments performed.
